# P-1537. *Staphylococcus aureus* Cell Wall Modifications Regulate Innate Immunity By Modulating Activation of Cytosolic DNA Sensors During Noncytotoxic Infection

**DOI:** 10.1093/ofid/ofaf695.1718

**Published:** 2026-01-11

**Authors:** Jordan Jastrab, Jonathan Kagan, Daniel Fisch

**Affiliations:** Brigham and Women's Hospital, Boston, Massachusetts; Boston Children's Hospital, Boston, Massachusetts; Boston Children's Hospital, Boston, Massachusetts

## Abstract

**Background:**

*Staphylococcus aureus* (*S. aureus*) causes persistent infections during which bacteria reduce toxin production and evade host immunity to survive. The inflammatory cytokine IL-1ß, which is released upon activation of immune complexes called inflammasomes, coordinates immunity to acute *S. aureus* infections. It is unclear how S. aureus evades IL-1ß-driven immunity during persistent infections.

**Figure 1.**
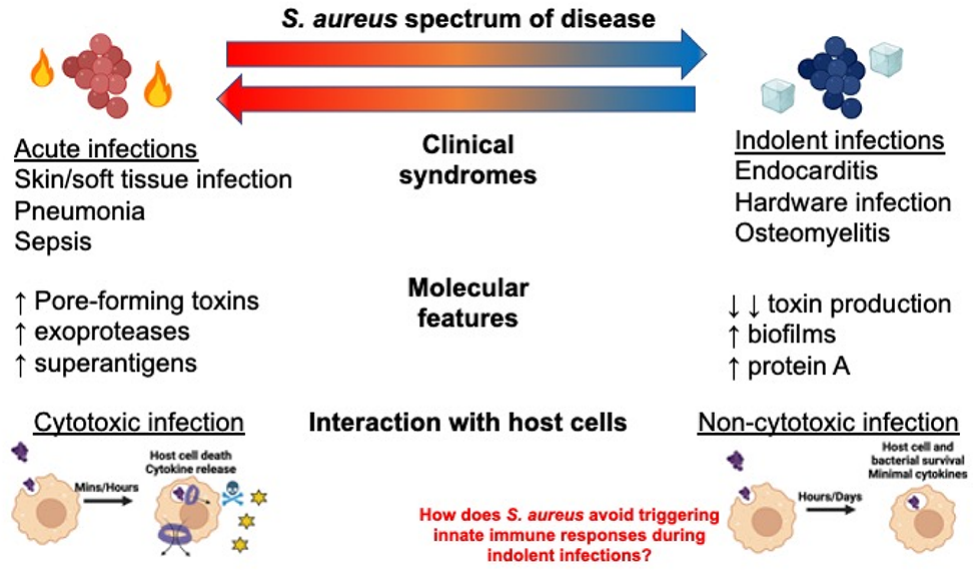
The double life of Staphylococcus aureus S. aureus can cause infections a wide variation of clinical characteristics. Indolent S. aureus infections are associated with strains that produce minimal toxins and that cause non-cytotoxic infections of host cells with minimal associated cytokine release.

**Figure 2.**
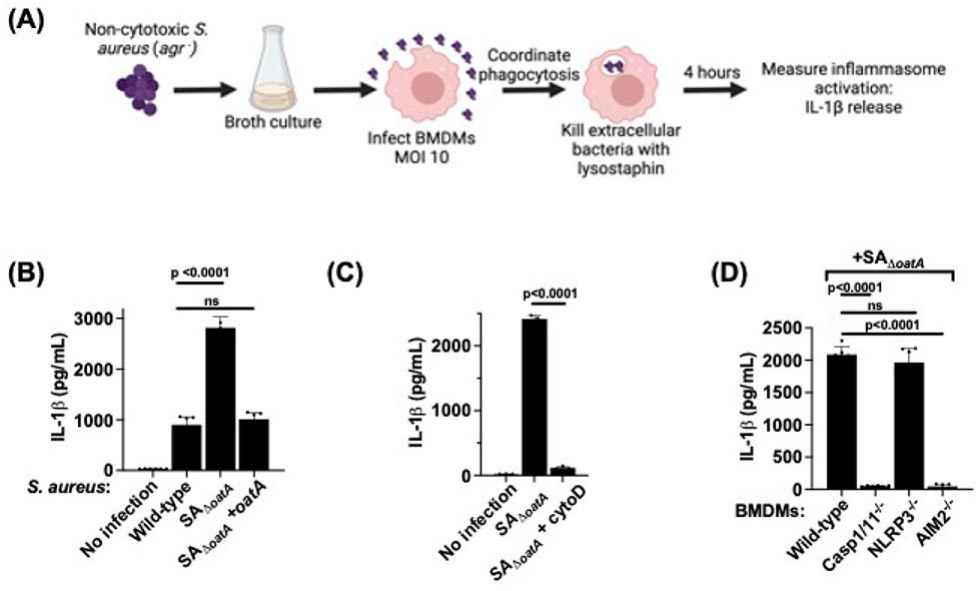
S. aureus peptidoglycan O-acetylation blunts activation of the AIM2 inflammasome (A) Schematic depicting infection model. BMDM: murine bone marrow-derived macrophage; MOI: multiplicity of infection; IL-1B: interleukin-1B. (B) Wild-type (WT) BMDMs were infected with the indicated S. aureus strains. (C) WT BMDMs were infected with S. aureus oatA with or without phagocytosis inhibitor cytochalasin D (CytoD); lysostaphin was not added for this experiment. (D) BMDMs generated from the indicated mouse lines deficient in inflammasome components were infected with S. aureus oatA. For all experiments, statistical significance was assessed by Student's t-test.

**Methods:**

We infected murine bone marrow-derived macrophages (BMDMs) with noncytotoxic *S. aureus* strains to simulate host-pathogen interactions during persistent infections. We evaluated cytokine production with immunoassays, bacterial killing by enumerating live intracellular bacteria, phagocytosis using microscopy, and quantified bacterial DNA using quantitative PCR. We generated mutant BMDM lines and *S. aureus* strains to identify host and bacterial factors that impact macrophage responses to infection.

**Figure 3.**
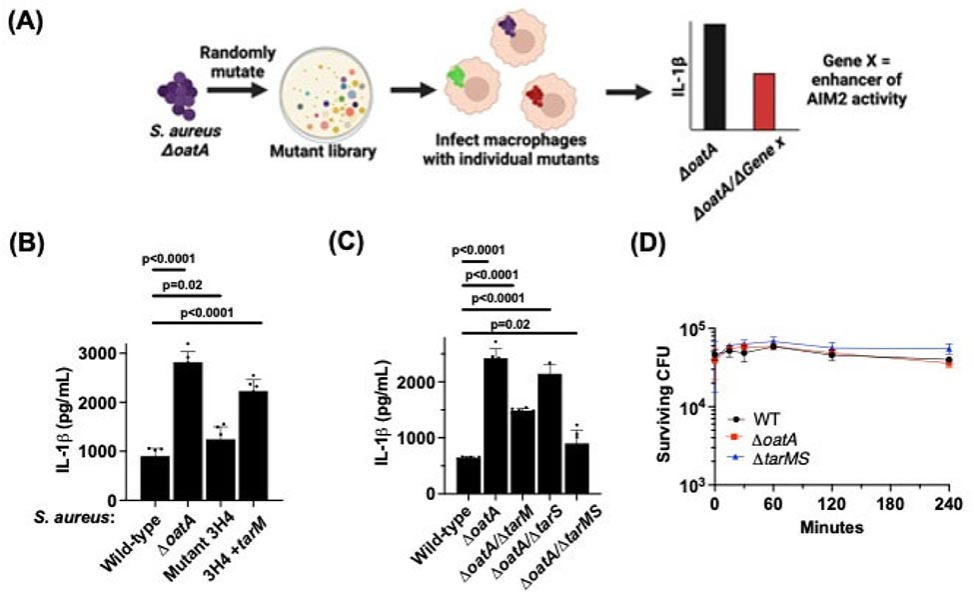
Wall teichoic acid glycosylation enhances AIM2 activation independent of bacterial survival (A) Schematic depicting the design of a suppressor screen to identify S. aureus genes that promote AIM2 activation during infection. (B,C) WT BMDMs were infected with the indicated S. aureus strains. 3H4: mutant strain identified by suppressor screen. (D) WT BMDMs were infected with the indicated S. aureus strains at an MOI=1, and at the indicated timepoints extracellular bacteria were washed and surviving attached/internalized bacteria were quantified.

**Figure 4.**
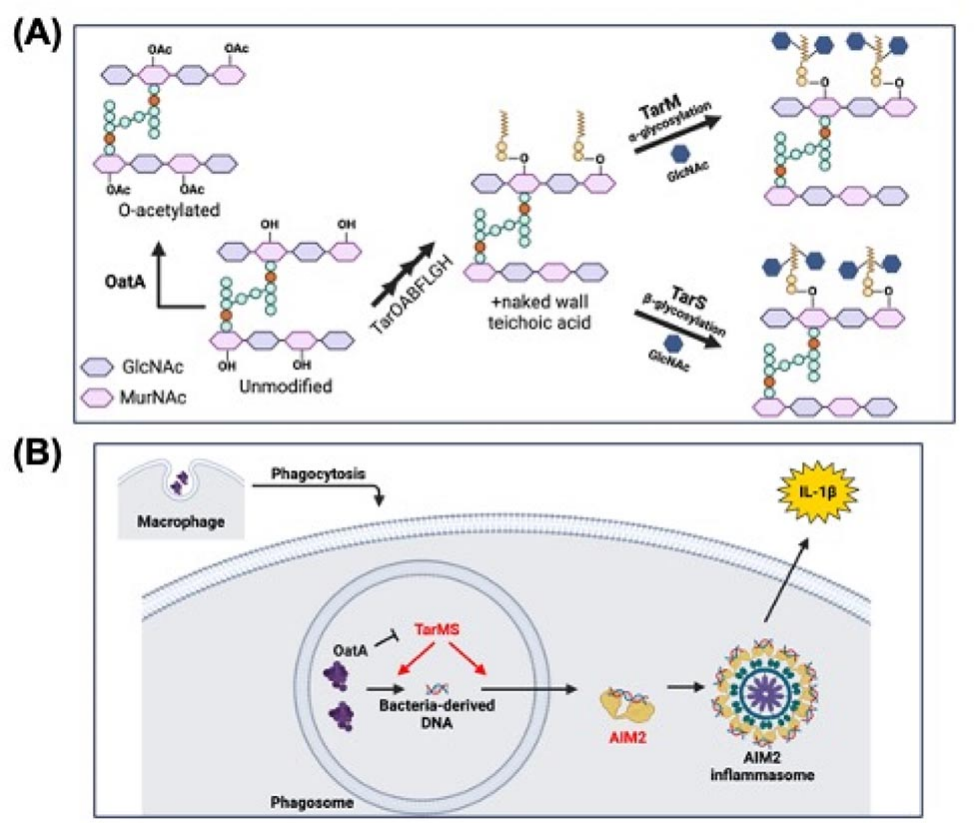
S. aureus cell wall modifications competitively modulate AIM2 activity to control the innate immune response to infection. (A) Cartoon depicting S. aureus peptidoglycan (PGN) and the relationship between PGN O-acetylation, wall teichoic acid (WTA) addition, and WTA glycosylation. GlcNAc: N-acetylglucosamine; MurNAc: N-acetyl muramic acid. (B) Cartoon depicting the proposed roles of of OatA and WTA glycosyltransferases in controlling the innate immune response to non-cytotoxic S. aureus infection.

**Results:**

*S. aureus* strains lacking peptidoglycan (PGN) O-acetyltransferase OatA (SA_Δ_*_oatA_*) triggered increased IL-1ß release compared to wild-type (WT) bacteria in a phagocytosis-dependent manner. IL-1ß release required macrophage AIM2, a cytosolic receptor that triggers inflammasome formation upon binding DNA. Using a transposon mutagenesis screen, we discovered wall teichoic acid (WTA) glycosyltransferases TarMS drive increased AIM2 activity. OatA reduced and TarMS increased both availability of bacterial DNA within macrophages and activation of another cytosolic DNA sensor, cGAS. OatA and TarMS did not impact internalization into or survival within macrophages.

**Conclusion:**

Our data reveal competing roles for *S. aureus* cell wall modifications in modulating immunity during noncytotoxic infection. OatA blunts, whereas TarMS enhance, availability of bacterial DNA and activation of cytosolic DNA sensors. These findings suggest *S. aureus* cell wall modifications manipulate immunity by altering availability of bacterial DNA within host immune cells. Further study should elucidate the molecular mechanisms by which *S. aureus* cell wall modifications contribute to DNA-driven immunity and may suggest new strategies for the development of immune-based therapies for persistent *S. aureus* infection.

**Disclosures:**

Jordan Jastrab, MD, PhD, Moderna: Stocks/Bonds (Public Company) Jonathan Kagan, PhD, Corner Therapeutics: Board Member|Corner Therapeutics: salary|Corner Therapeutics: Ownership Interest|Corner Therapeutics: Stocks/Bonds (Private Company)|Larkspur: Advisor/Consultant|Larkspur: Ownership Interest|Larkspur: Stocks/Bonds (Private Company)|MindImmune: Advisor/Consultant|MindImmune: Ownership Interest|MindImmune: Stocks/Bonds (Private Company)|Neumora: Advisor/Consultant|Neumora: Ownership Interest|Neumora: Stocks/Bonds (Private Company)

